# A novel system for stable, high-level expression from the T7 promoter

**DOI:** 10.1186/1475-2859-11-109

**Published:** 2012-08-16

**Authors:** Malgorzata Kesik-Brodacka, Agnieszka Romanik, Diana Mikiewicz-Sygula, Grazyna Plucienniczak, Andrzej Plucienniczak

**Affiliations:** 1 Department of Bioengineering, Institute of Biotechnology and Antibiotics, Staroscinska 5, Warsaw, 02-516, Poland

**Keywords:** T7 expression, Expression cassette, Stable expression from T7 promoter, High expression from T7 promoter

## Abstract

**Background:**

The most widespread, efficient prokaryotic protein-producing system is one where the T7 phage polymerase recognizes the T7 phage promoter (T7 p/p system). Unfortunately, in this system, target protein expression gradually declines and is often undetectable following 3 to 5 subcultures. Although a number of studies have attempted to stabilize the expression levels of the T7 p/p system, none has resolved the problem adequately and thus precludes the use of this system for the production of recombinant proteins on a large scale.

**Results:**

We created an expression cassette enabling stable, high-level expression in the T7p/p system. The cassette was tested with two different vector backbones and two target proteins. In all experiments, the expression system using the new cassette exhibited high and stable protein expression levels when compared to the traditional system.

**Conclusions:**

Herein, we describe a universal expression cassette that enables high-level, stable target protein expression in T7 RNA polymerase-based expression systems. We also present the successful use of this cassette as a novel expression platform and demonstrate its ability to overcome the main deficiency of the T7 p/p system. Thus, we provide a method for using the T7 p/p system on an industrial scale.

## Background

The potential to produce recombinant proteins in prokaryotic systems, which are difficult and/or expensive to enlist from natural sources, was a major breakthrough in biotechnology [1]. It was enabled by a discovery in the 1980's, in which a system based on RNA polymerases, dependent upon DNA, was incorporated into host cells [2]. These cells were transformed with vectors bearing a recombinant gene under the control of promoters recognized by these polymerases. Currently, one of the most widely used prokaryotic protein-producing systems uses the T7 phage polymerase that recognizes the T7 phage promoter [3]. In many cases, this system has proven extremely effective and has permitted high levels of target protein expression to be obtained, up to 50% of the total cellular protein production [4]. However, the system is not without its problems, especially a gradual decrease in the expression levels of the target gene. After several (3 to 5) subcultures, the induced expression levels may be comparable to basal expression levels, and often the host cells do not express detectable levels of the target protein [5]. This results in a lower target protein yield. Previously, it was thought that decreased protein expression was caused by loss of, or mutations in, the plasmid bearing the target gene. Many attempts have been made to stabilize the expression levels of the T7 p/p system, including examination of plasmid stability by lowering the uninduced T7 polymerase production levels. Another approach used freshly transformed bacteria to obtain the target protein [5-9]. However, none of these approaches have resolved the problem adequately.

The aim of our study was to address the deficiencies of traditional T7 promoter-based systems and create a novel system enabling long-term, stable expression of target proteins in bacteria cell factories.

The basis for our strategy was that the major contributing factor of decreased expression levels in the T7-based system was random chromosomal mutations occurring in the sequence encoding the T7 phage RNA polymerase. Thus, selection pressure favored cells whose metabolism was not burdened by the production of the target protein [5]. In traditional systems, non-overproducing cells quickly dominate the culture [10]. Our approach aims to overcome the problem of reduced expression by eliminating non-overproducing cells from the culture. We achieved this goal by the creation of a stable expression T7C p/p system in which the loss of functional polymerase, T7 RNA polymerase promoter, plasmid and any failure disabling the production of the target recombinant protein induces the death of the host cell. Therefore, we maintain only target protein-expressing cells in the culture and achieve a stable high level of recombinant protein expression.

## Results and discussions

**Figure 1:**
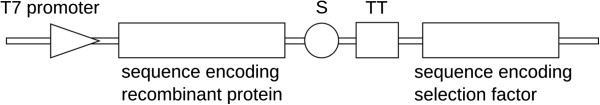
**Schematic representation of the expression cassette with the T7 promoter.** The diagram represents a variant of the cassette. TT–transcription terminator sequence from the tryptophan operon; S–translation termination codon (TAA).

The novel T7C p/p system created was based on an expression cassette (ECKm cassette) (Figure 1) controlled by a T7 promoter introduced into the expression vector. The gene encoding aminoglycoside-3’-phosphotransferase (APH) that induces kanamycin resistance, was inserted to the ECKm cassette as a selection factor. A transcription terminator for promoters other than the T7 phage promoter was placed ahead of the 5' end of the selection marker APH gene to preclude the expression of the selection marker by any polymerase other than the T7 phage polymerase. This prevented expression of the selection marker by non-phage promoters present in the expression vector, which may be recognized by the host cell polymerases. Therefore, production of the selection marker was strictly controlled by the T7 phage promoter recognized by the T7 phage polymerase. This resulted in the novel cassette exhibiting the required characteristics.

**Figure 2:**
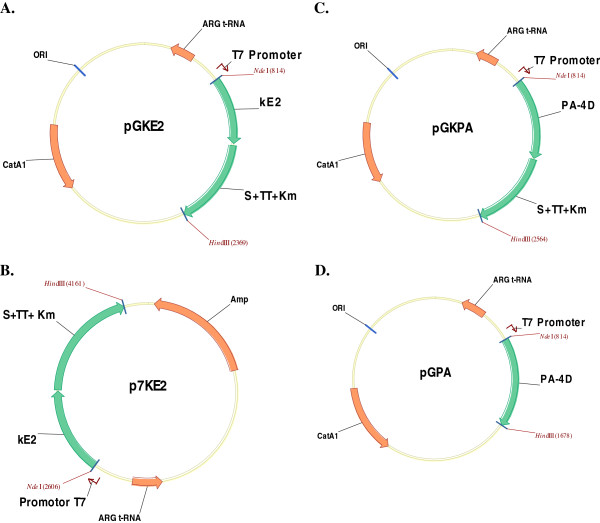
**Expression vector maps.** ARG t-RNA−gene encoding AGA and AGG tRNA; CatA1−chloramphenicol resistance gene; Amp−ampicillin resistance gene; ORI−origin of replication; PA-4D−the fourth domain of the protection antigen of *Bacillus anthracis*. Target protein; kE2−fragment of the E2 antigen of CSFV. Target protein; S + TT + Km−expression cassette.

We tested the function of the expression cassette when introduced into two plasmid backbones, pIGDMCT7RS and pT7RS, using two different target polypeptides, the kE2 protein from the Classical Swine Fever Virus and the PA-4D protein from *Bacillus anthracis* (Figure 2). Both plasmid vectors used contained genes encoding AGA and AGG tRNAs, which supplement the shortage of these tRNAs that results from the codon usage in *Escherichia coli*. We used two types of selection markers in our experiments. The Type I selection marker is functionally independent from the expression cassette, and is present on the expression vector and forces the preservation of the vector in the host cells when they are cultured in the selective medium. This procedure is standard for cell selection, and thus we could replicate the behavior of a standard T7 p/p system in which only cells that do not express the selection marker are eliminated from the culture.

The Type II selection marker was tightly linked to the expression of the target protein. This type of marker could be expressed only when all elements of the T7 phage promoter-based system remained functional. As a result, from the earliest culture stages, any damage that impaired a cell's ability to produce the target protein also caused it to stop producing the selection marker. Therefore, only those cells expressing the target protein were able to grow in the culture. The Type II selection marker is an integral part of the expression cassette.

To confirm that T7 polymerase is required and responsible for the expression of selection marker from the cassette we performed experiment A. In this experiment we transformed Bl21(DE3) and DH5α *E. coli* strains with plasmids containing the expression cassette. Bl21(DE3) is a λDE3 lysogen strain of *E. coli*, which harbors a genomic copy of the gene for T7 RNA polymerase under the control of the *lac* repressor. In this strain, T7 RNA polymerase is produced after induction with isopropyl-β-D-galactoside (IPTG).

DH5α, the second strain used in the experiment, has no T7 bacteriophage polymerase gene in its chromosome. Both bacteria strains were transformed with pGKE2, pGKPA and p7KE2 plasmids. The expression vectors used in the experiment are listed in Table 1 and shown in Figure 2. Bacteria transformed with pGKE2 and pGKPA were plated on LB medium supplemented with Cm or Km while bacteria transformed with p7KE2 were plated on LB supplemented with Amp or Km.

**Table 1:** Construction of the expression vectors

**Expression vector name**	**Plasmid backbone**	**Expression cassette**	**Sequence encoding target protein**
pGKE2	pIGDMCT7RS	ECKm	kE2 gene of CSFV virus.
pGKPA	pIGDMCT7RS	ECKm	PA-4D of *B. anthracis*
pGPA	pIGDMCT7RS	(none)	PA-4D of *B. anthracis*
p7KE2	pT7RS	ECKm	kE2 gene of CSFV virus.

*E. coli* DH5α transformed with the plasmids were growing normally when plated on LB supplemented with Cm or Amp. However, bacteria of this strain did not grow on LB supplemented with Km. Type I selection marker independent from T7 polymerase is responsible for resistance to Cm and Amp antibiotics. On the other hand, resistance to Km is granted by Type II selection marker and is dependent on the presence of polymerase T7. As DH5α does not express the T7 polymerase, there is also no expression of Type II selection marker.

*E. coli* Bl21(DE3) transformed with appropriate plasmid grew on LB supplemented with Cm, Amp and Km. In our experiments it was observed, that even without induction with IPTG the basal expression level of T7 polymerase was sufficient to grant bacteria resistance to Km, as the cells grew in presence of Km without the IPTG induction.

The results from this experiment prove that the cassette exhibits the required characteristics: the bacteria are resistant to Km only when the T7 polymerase is produced.

Subsequently, we performed a series of experiments to characterize the new expression system. Table 2 summarizes the design of experiments described below. The expression vectors used in the experiments are listed in Table 1 and shown in Figure 2.

**Table 2:** Experimental design

**Experiment**	**Expression vector**	**Markers**		**Type of experiment**	**Antibiotic in selective medium**	**Target protein**
		**Type I**	**Type II**			
B	pGKE2	CmR	KmR	♦ experiment	Km	kE2
				◊ control	Cm	
C	p7KE2	AmpR	KmR	♦ experiment	Km	kE2
				◊ control	Amp	
D	pGKPA	CmR	KmR	♦ experiment	Km	PA-4D
	pGPA	CmR	−	◊ control	Cm	

*E. coli* strain, BL21(DE3), was used for the overproduction of proteins in all the experiments.

**Figure 3:**
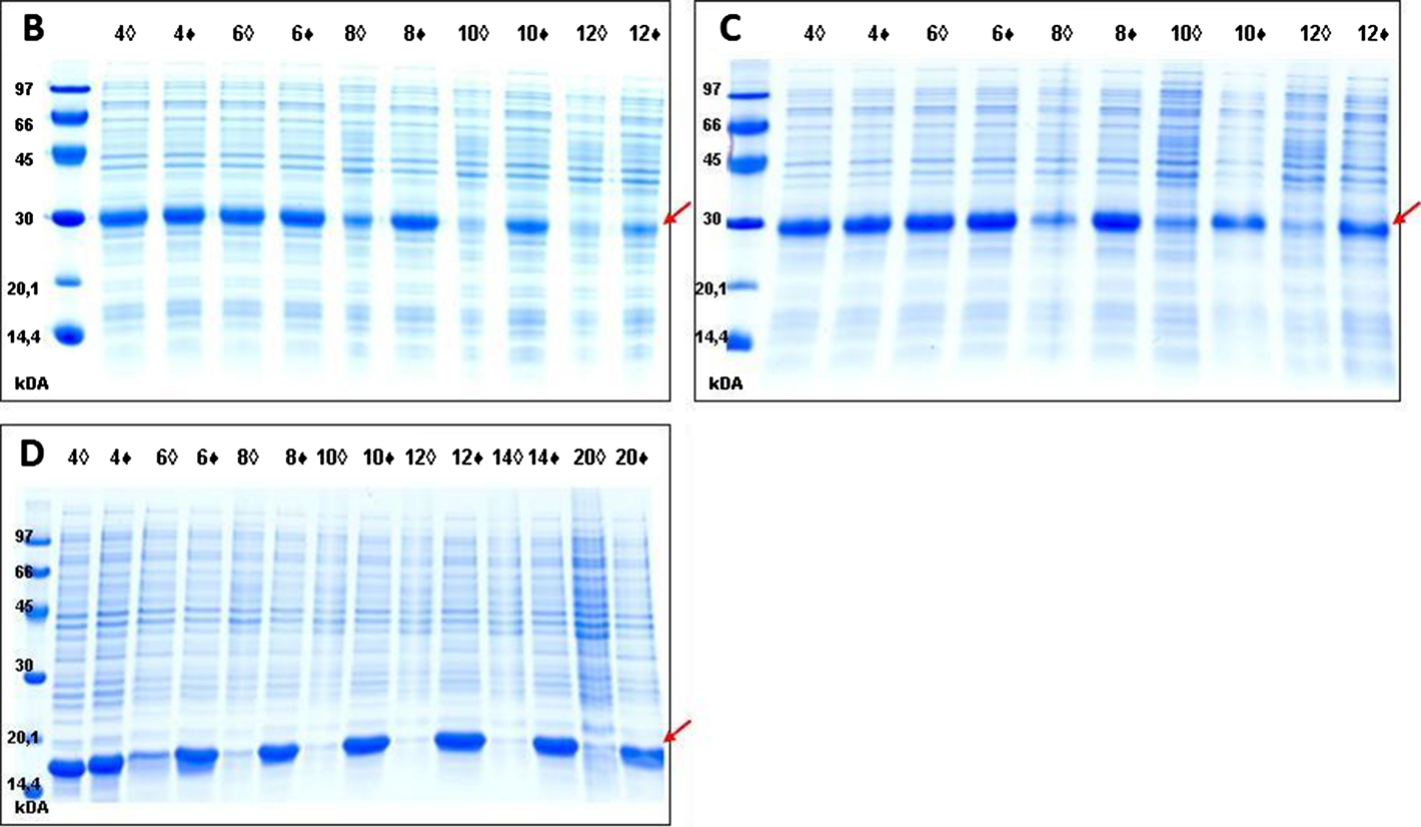
**Comparison of the expression levels of target proteins in *E. coli* with or without the selection of expressing cells.** The SDS-PAGE results in B, C and D show the expression levels of the target protein from experiments B, C and D respectively. The numbers over each lane indicate how many hours passed between the induction with IPTG and the collection of the sample. ♦–experimental cultures: selection with kanamycin; ◊–control cultures: medium without kanamycin.

Experiment B examined the difference in the expression levels of the kE2 protein in *E. coli* harboring the pGKE2 expression vector between cultures with different selection markers: chloramphenicol resistance (CmR) as the Type I marker and kanamycin resistance (KmR) as the Type II marker. Results of these experiments are presented in Figure 3B.

Experiment C analyzed the difference in expression levels of the kE2 protein in *E. coli* harboring the p7KE2 expression vector between cultures with different selection markers: ampicillin resistance (AmpR) as the Type I marker and KmR as the Type II marker. Results of these experiments are shown in Figure 3C.

Experiment D compared the expression levels of the target polypeptide obtained in a commonly used system, T7 p/p, with the expression levels for the novel T7C p/p system described here. To this end, two vectors were used, a standard vector with the T7 phage promoter and an introduced sequence encoding PA-4D (pGPA), and a modified vector containing the sequence encoding PA-4D as a part of the expression cassette (pGKPA). We used two different selection markers: CmR as the Type I marker and KmR as the Type II marker. Results of these experiments are presented in Figure 3D.

Target protein expression levels were the same for both selection marker types between 4 to 6 hours after induction (Figure 3B,C,D). However, at 6 to 8 hours after protein expression induction, cultures with the Type I selection marker demonstrated diminished target protein expression levels. This phenomenon was observed in all three experiments (B, C and D). The gradual decrease in expression levels of the target gene is because of the Type I selection that forces the cell to retain the expression vector and protect the expression mechanism of the selection marker but not the target protein. Because overexpression of the target protein poses a significant burden on the host cell metabolism, cells that undergo mutations that allow the expression of the selection marker, but not the target protein, are favored, divide faster, and ultimately overgrow the culture.

In the experimental cultures with the Type II marker, we observed consistently high expression levels throughout the course of the experiments. Notably, the observed protein expression levels for this marker type were equal to initial protein expression levels observed in experiments using Type I marker. While the expression levels were stable for Type II marker, they were visibly declining for Type I marker.

Differences in expression levels between cultures with the Type I and Type II markers increased with time and were caused by the Type II marker allowing to survive only these cells which maintained the expression vector and had a correctly functioning expression system. This eliminated non-overproducer cells from the culture.

Our results show that improved stability did not impair the target protein expression levels. In addition the production level of the Type II selection factor is so low, that it is not seen on SDS-PAGE (Figure 3). Yet, as our experiments demonstrate, the expression level of the selection marker was sufficient to allow the host cells to grow and divide in the selection media. Additionally, our system exploited the phenomenon of the basal expression of T7 RNA polymerase, allowing medium containing the selective antibiotic to be used at all stages of the culture (even before induction). In traditional T7 p/p systems, the existence of even basal expression generates a burden on the host cells metabolism, giving a selective advantage to non-overproducing cells [11].

The T7C p/p system can also be used to express proteins toxic to *E. coli* cells. However, in this case basal expression is disadvantageous, and specialized commercially available strains expressing T7 lysozyme should be used [7]. In addition, this case requires the selective antibiotic to be added to the medium after induction of T7 RNA polymerase production (data not shown).

The described T7C p/p system allows optimization of the target protein expression, indicating the possibility of obtaining high yields of the target protein in large-scale cultures. High target protein content at the time of culture harvest facilitates target protein purification.

An additional advantage of T7C system is the reduction of time-consuming laboratory procedures. Small-scale test expression is widely used as a predictive tool to determine which of the clones produces the target protein [3]. Use of T7C system allows omission of this test by culturing of bacteria in the presence of an antibiotic from the earliest stage (even before induction). Therefore eliminating non-overproducing cells from the culture. The gain is especially notable when testing large numbers of genetic constructs for target protein expression.

## Conclusions

In this study, we present a novel, universal method for stable, high-level recombinant protein production in bacterial cells. This method increases the efficiency and cost competitiveness of target protein production in bacterial cells. We demonstrate the applicability of this method for the enhancement of the T7 phage RNA polymerase-based expression system, the most widespread recombinant protein production system used in laboratories worldwide. We demonstrated the ability of this system to overcome the chief deficiency of the T7p/p system – the gradual decline of target protein production. We tested the method using two different vector backbones and two different target proteins. In all experiments, stable, high-level long-term expression of the target protein was attained.

This expression system can significantly enhance protein expression for both the laboratory and large-scale applications, and its use facilitates purification of recombinant proteins.

## Methods

### ECKm cassette construction

A schematic representation of the expression cassette with the T7 promoter is shown in Figure 1. The sequences of the oligonucleotides and primers used for construction of the cassettes and the expression vectors are listed in Table 3. All expression vectors used are listed in Table 1.

**Table 3:** Primers used for cassettes and vector construction

**Primer name**	**Sequence (5′ → 3′)**
SalINotI 5'Kan	GGGGTCGACGCGGCCGCAAGGGGTGTTATGAGCCA
HindIII 3'Kan	AAAAGCTTAGAAAAACTCATCGAGCA
Tryp1	TCGACCTAAGCGGCCGCTAATCCCACAGCCGCCAGTTC
Tryp2	CGCTGGCGGCATTTT
Tryp3	GCGGAACTGGCGGCTGTGGGATTAGCGGCCGCTTAGG
Tryp4	GGCCAAAATGCCGCCA
NdeI 5'E2	GAGGGCATATGGCACGTCTAGCCTGCAAGGAAGAT
XhoI 3'E2	AAAAGCTTCTCGAGTTAGTAGTGTGGGAGTCCGTCAG
NdeI 5'PA4D	GGGGATTCATATGAAACGTTTTCATTATGATCGCAATAAC
SalI 3'PA4D	AAAAGCTTCTCGAGTTATCCTATCTCATAGCCTTTTTTAG
HindIII 3'PA4D	AAAAGCTTATCCTATCTCATAGCCTTTTTTAG

Four synthetic oligonucleotides (Tryp1, Tryp2, Tryp3, and Tryp4) were ligated to form a synthetic DNA fragment encoding the transcription terminator sequence of the tryptophan operon. A TAA translational stop codon (designated S), *Sal*I "sticky" end and *Not*I restriction site were added to the 5’-end of the fragment. A *Not*I "sticky" end was introduced at the 3’-end of the fragment. The created DNA fragment was designated S + TT.

The DNA fragment containing the region encoding aminoglycoside-3’-phosphotransferase (APH) (kanamycin resistance gene) and 10 nt of non-coding sequence upstream of the gene was amplified by PCR with *Sal*I*Not*I 5'Kan and *Hin*dIII 3'Kan primers. *Sal*I and *Not*I restriction sites were added to the 5’-end. A *Hin*dIII restriction site and a TAA translation termination codon were introduced at the 3’-end of the amplified fragment. The PCR fragment was digested with *Sal*I and *Hin*dIII.

The digested S + TT DNA fragment and the APH gene fragment were ligated together to create the ECKm cassette.

### Expression vector construction

To clone the expression cassettes, we used two plasmid backbones: pIGDMCT7RS and pT7RS [GenBank accession nos. DQ485721 and AY923866, respectively]. These vectors carry a T7 phage RNA polymerase promoter, a sequence encoding the translational stop codon of the T7 phage and genes encoding AGA and AGG tRNAs, which supplement the shortage of these tRNAs that results from the codon usage in *E. coli*. Both plasmids carry resistance genes, allowing the selection of bacteria harboring these plasmids. pIGDMCT7RS carries a chloramphenicol resistance gene, CatA1, and pT7RS carries an ampicillin resistance gene.

The ECKm cassette was cloned into the pIGDMCT7RS and pT7RS backbones between the *Sal*I and *Hin*dIII restriction sites, and the resulting plasmids were designated pGK and p7K, respectively.

For target proteins, two different polypeptides originating from two organisms were used: a 220 amino acid (aa) fragment of the E2 (kE2) protein from Classical Swine Fever Virus (CSFV) and PA-4D (143 aa) of *Bacillus anthracis*.

The complete nucleotide sequence of the E2 antigen of CSFV was used to amplify kE2, a 1023 bp fragment corresponding to nt 2428–3087, according to the E2 sequence [GenBank accession no. M31768]. The sequence was amplified by PCR with *Nde*I 5'E2 and *Xho*I 3'E2 primers. An *Nde*I site and GCA codon were added, and the guanine was altered to a thymine at the 5’-end. A *Hin*dIII restriction site and TAA translation stop codon were introduced at the 3’-end of the amplified fragment. The kE2 fragment was digested with *Nde*I and *Hin*dIII restriction enzymes and cloned into pGK using the same restriction sites. The resulting vector was designated pGKE2 (Figure 2A).

To create p7KE2 (Figure 2B), a segment consisting of the sequences encoding kE2, the transcription terminator sequence from the tryptophan operon, and APH was removed from the pGKE2 vector using *Nde*I and *Hin*dIII restriction enzymes and shuttled into the pT7RS backbone at the *Nde*I-*Hin*dIII restriction sites.

The complete nucleotide sequence of the protection antigen (PA) of *Bacillus anthracis* was used to amplify the fourth domain (PA-4D) of PA, a 429 bp fragment, according to the PA sequence [GenBank accession no. AF065404]. Two amplifications were conducted: the first one with *Nde*I 5'PA4D and *Sal*I 3'PA4D primers; the second with *Nde*I 5'PA4D and *Hin*dIII 3'PA4D primers. Because of the first amplification, an *Nde*I restriction site was added at the 5’-end, and a *Sal*I restriction site was added at the 3’-end of the amplified fragment. In the second amplification, an *Nde*I restriction site was added at the 5’-end, and a *Hin*dIII restriction site was added at the 3’-end of the amplified fragment. The amplified fragment was then digested with *Nde*I and *Sal*I and cloned into the pGK vector at the corresponding restriction sites. The final vector was designated pGKPA (Figure 2C). The fragment digested with *Nde*I and *Hin*dIII was cloned into the *Nde*I/*Hin*dIII restriction sites of pIGDMCT7RS. The final vector was designated pGPA (Figure 2D).

All described vectors are summarized in Table 1.

Standard protocols were used for PCR, restriction digests, ligations, and transformations as previously described [12,13].

### Expression of selection markers

In the experiments were used competent DH5α and Bl21(DE3) *E. coli* strains purchased form Stratagene. Bacteria were transformed with plasmids pGKPA, pGKE2, p7KE2. All these plasmids contain ECKm expression cassette. After the transformation bacteria were plated in duplicate onto LB medium supplemented with appropriate antibiotic: ampicillin-(100 mg/l), chloramphenicol-(18 mg/l), kanamycin-(25 mg/l).

### Expression of the target protein using expression vectors with the ECKm cassette

The *Escherichia coli* BL21(DE3) strain was used for overproduction of recombinant proteins in all experiments. The bacteria strain was obtained from Stratagene. *E. coli* bacteria harboring the appropriate recombinant plasmid were grown in 3 ml of Luria Bertani medium (10 g/L tryptone, 10 g/L NaCl, and 5g/L yeast extract) at 37°C for 3 hours. The cultures were then diluted with fresh medium (1:100) and shaken at 37°C until the A_600_ reached 0.4–0.7. Subsequently, the target polypeptide expression was induced by the addition of IPTG (0.1 mM final concentration), and the shaking was continued for 12 to 20 hours. Media used in the experiments contained the appropriate antibiotic: kanamycin (25 mg/l), chloramphenicol (18 mg/l), or ampicillin (100 mg/l).

Samples for A_600_ measurements and electrophoretic separation on polyacrylamide gels (SDS-PAGE) were collected every 2 hours after induction.

### Protein analysis

The presence and quantity of the target polypeptide was determined by separation of the bacterial lysate on a 15% polyacrylamide gel (SDS-PAGE), carried out as described by Laemmli [14]. The separated proteins were visualized by staining with Coomassie Brilliant Blue G.

## Abbreviations

Km = Kanamycin; Amp = Ampicillin; Cm = Chloramphenicol; PCR = Polymerase chain reaction; Nt = Nucleotide; Aa = Amino-acid residue; Bp = Base pair(s); IPTG = Isopropyl-β-D-galactoside; SDS = Sodium dodecyl sulphate.

## Competing interests

The authors declare no competing financial interests.

## Authors' contributions

MKB and AP designed and performed all of the experiments. MKB, AR and AP analyzed the data. DMS and GP provided the plasmid backbones. MKB wrote the manuscript. All the authors read and approved the final manuscript.

## References

[B1] Swartz JR Advances in Escherichia coli production of therapeutic proteins Curr Opin Biotechnol 2001 12 195 201 10.1016/S0958-1669(00)00199-3 11287237

[B2] Studier FW Rosenberg AH Dunn JJ Dubendorff JW Use of T7 RNA polymerase to direct expression of cloned genes Methods Enzymol 1990 185 60 89 2199796 10.1016/0076-6879(90)85008-c

[B3] Gräslund S Protein production and purification Nat Methods 2008 5 135 146 10.1038/nmeth.f.202 18235434 PMC3178102

[B4] Jevsevar S Gaberc-Porekar V Fonda I Podobnik B Grdadolnik J Menart V Production of nonclassical inclusion bodies from which correctly folded protein can be extracted Biotechnol Prog 2005 21 632 639 15801811 10.1021/bp0497839

[B5] Vethanayagam JG Flower AM Decreased gene expression from T7 promoters may be due to impaired production of active T7 RNA polymerase Microb Cell Fact 2005 4 3 10.1186/1475-2859-4-3 15638935 PMC545050

[B6] Saïda F Uzan M Odaert B Bontems F Expression of highly toxic genes in E. coli: special strategies and genetic tools Curr Protein Pept Sci 2006 7 47 56 10.2174/138920306775474095 16472168

[B7] Studier FW Use of bacteriophageT7 lysozyme to improve an inducible T7expression system J Mol Biol 1991 219 37 44 10.1016/0022-2836(91)90855-Z 2023259

[B8] Jana S Deb JK Strategies for efficient production of heterologous proteins in Escherichia coli Appl Microbiol Biotechnol 2005 67 289 298 10.1007/s00253-004-1814-0 15635462

[B9] Mertens N Remaut E Fiers W Tight transcriptional control mechanism ensures stable high-level expression from T7 Promoter-based expression plasmids Nat Biotechnol 1995 13 175 179 10.1038/nbt0295-175 9634760

[B10] Tyo KE Ajikumar PK Stephanopoulos G Stabilized gene duplication enables long-term selection-free heterologous pathway expression Nat Biotechnol 2009 27 760 765 10.1038/nbt.1555 19633654

[B11] Kelley KC Huestis KJ Austen DA Sanderson CT Donoghue MA Stickel SK Kawasaki ES Osburne MS Regulation of sCD4-183 gene expression from phage-T7-based vectors in Escherichia coli Gene 1995 156 33 36 10.1016/0378-1119(95)00008-T 7737513

[B12] Sambrook J Russell DW Molecular Cloning A Laboratory Manual 2001 Cold Spring Harbor New York: Cold Spring Harbor Laboratory Press

[B13] Ausubel FM Brent R Kingston RE Moore DD Seidman JG Smith JA Struhl K Current Protocols in Molecular Biology 1994 Brooklyn, New York: John Wiley and Sons, Inc

[B14] Laemmli UK Cleavage of structurals proteins during the assembly of the head of bacteriophage T4 Nature 1970 227 680 685 10.1038/227680a0 5432063

